# Stepwise enhancement of catalytic performance of haloalkane dehalogenase LinB towards β-hexachlorocyclohexane

**DOI:** 10.1186/s13568-014-0072-5

**Published:** 2014-09-21

**Authors:** Ryota Moriuchi, Hiroki Tanaka, Yuki Nikawadori, Mayuko Ishitsuka, Michihiro Ito, Yoshiyuki Ohtsubo, Masataka Tsuda, Jiri Damborsky, Zbynek Prokop, Yuji Nagata

**Affiliations:** 1 Department of Environmental Life Sciences, Graduate School of Life Sciences, Tohoku University, Sendai, Japan; 2 Loschmidt Laboratories, Department of Experimental Biology and Research Centre for Toxic Compounds in the Environment RECETOX, Faculty of Science, Masaryk University, Kamenice 5/A13, Brno, 625 00, Czech Republic; 3 The United Graduate School of Agricultural Science, Gifu University 1–1 Yanagido, Gifu 501-1193, Japan; 4 Consolidated Research Institute for Advanced Science and Medical Care, Waseda University, 2-2 Wakamatsu-cho, Shinjuku, Tokyo 162-8480, Japan

**Keywords:** β-Hexachlorocyclohexane, Xenobiotics, Biodegradation, Haloalkane dehalogenase, Protein evolution

## Abstract

Two haloalkane dehalogenases, LinB_UT_ and LinB_MI_, each with 296 amino acid residues, exhibit only seven amino acid residue differences between them, but LinB_MI_’s catalytic performance towards β-hexachlorocyclohexane (β-HCH) is considerably higher than LinB_UT_’s. To elucidate the molecular basis governing this difference, intermediate mutants between LinB_UT_ and LinB_MI_ were constructed and kinetically characterized. The activities of LinB_UT_-based mutants gradually increased by cumulative mutations into LinB_UT_, and the effects of the individual amino acid substitutions depended on combination with other mutations. These results indicated that LinB_UT_’s β-HCH degradation activity can be enhanced in a stepwise manner by the accumulation of point mutations.

## Introduction

γ-Hexachlorocyclohexane (γ-HCH; also known as γ-BHC or lindane) is a manmade and xenobiotic halogenated insecticide that was once used worldwide on a large scale. A number of soil bacterial strains that can aerobically degrade γ-HCH have been isolated from geographically distant locations (Lal et al. [2010]; Ito et al. [2007]; Lal et al. [2006]; Mohn et al. [2006]; Phillips et al. [2005]). As this novel compound was first released into the environment in the 1940s, they must have evolved quickly to utilize it.

**Figure 1:**
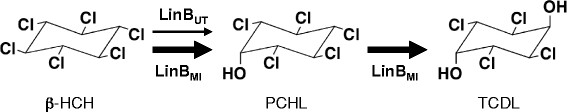
**β-HCH degradation reactions catalyzed by LinB**_**UT**_**and LinB**_**MI**_**.** LinB_MI_ converts β-HCH to PCHL and further to TCDL, while LinB_UT_ catalyzes only the first conversion step of β-HCH to PCHL.

An industrial chemical process of benzene photochlorination generates so-called technical-HCH (t-HCH), which consists mainly of five isomers, α- (60-70%), γ- (12-16%), β- (10-12%), δ- (6-10%), and ε-HCH (3-4%) (Vijgen et al. [2011]). Among these isomers, only γ-HCH has insecticidal activity; this isomer was therefore purified. The remaining isomers were in many cases improperly deposited, causing serious environmental problems. α- and β-HCH isomers as well as γ-HCH were categorized as persistent organic pollutants (POPs) at the Stockholm Convention (Vijgen et al. [2011]). Among the HCH isomers, β-HCH is the most recalcitrant; it is usually the predominant isomer remaining in contaminated soils and in animal tissues and fluids (Willett, et al. [1998]). All six chlorines of β-HCH in equatorial positions seem to contribute to its having the greatest chemical stability among the isomers. Several β-HCH-degrading bacterial strains have also been identified (Johri et al. [1998]; Gupta et al. [2000], [2001]). Haloalkane dehalogenase (HLD) LinB, which was originally described as an enzyme involved in γ-HCH degradation in *Sphingobium japonicum* UT26 (LinB_UT_) (Nagata et al. [1993]), was more recently identified as an enzyme possessing β-HCH degradation activity (Nagata et al. [2005]; Sharma et al. [2006]) (Figure 1).

**Figure 2:**
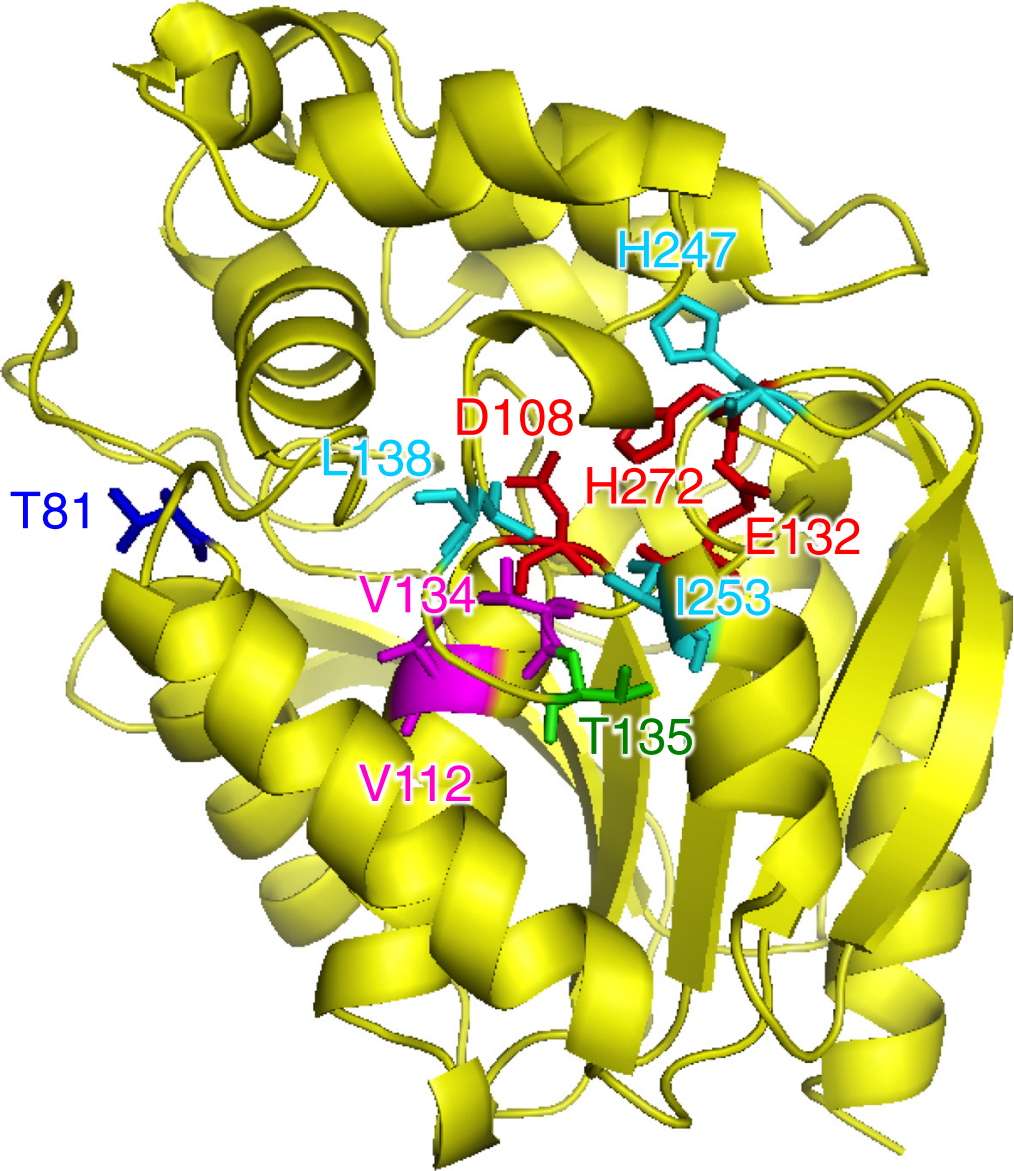
Structure of LinB_MI_ (PDB code 4H77) (Okai et al. [2013]) and location of catalytic triad (D108, E132, and H272; shown in red) and the seven dissimilar amino acid residues between LinB_MI_ and LinB_UT_: V134 and V112 (in magenta), L138, H247, and I253 (in cyan), T135 (in green), and T81 (in blue). See text for detail.

HLDs belong to the α/β-hydrolase family, and their catalytic mechanism consists of the following steps: substrate binding, cleavage of the carbon-halogen bond in the substrate and simultaneous formation of an intermediate covalently bound to a nucleophile, hydrolysis of the alkyl-enzyme intermediate, and release of halide ion and alcohol (Damborsky and Koca [1999]; Janssen [2004]; Prokop et al. [2003]). LinB_MI_ isolated from *Sphingobium* sp. MI1205 (Ito et al. [2007]) and LinB_UT_ each consist of 296 amino acid residues and share 98% sequence identity, with only seven different amino acid residues between them, at the positions 81, 112, 134, 135, 138, 247, and 253 (Figure 2). However, these two enzymes exhibit significantly different enzymatic behaviors in β-HCH degradation (Figure 1). LinB_MI_ catalyzes the two-step dehalogenation and converts β-HCH to 2,3,4,5,6-pentachlorocyclohexanol (PCHL) and then to 2,3,5,6-tetrachlorocyclohexane-1,4-diol (TCDL) (LinB_MI_-type activity) (Ito et al. [2007]), whereas LinB_UT_ catalyzes only the former step (Nagata et al. [2005]) (Figure 1). Furthermore, LinB_MI_ can catalyze the first conversion step an order of magnitude more rapidly than LinB_UT_ (Ito et al. [2007]). Substitution of the LinB_UT_ I134 and A247 residues, which form the catalytic pocket, to the LinB_MI_-type V and H residues, respectively, resulted in only a weak effect on LinB_MI_-type activity (Ito et al. [2007]). Additionally, the reciprocal double mutant of LinB_MI_ (V134I/H247A) still retained relatively high LinB_MI_-type activity (Ito et al. [2007]). These results indicated that one or more of the five other residues are also important for LinB_MI_-type activity. Our previous site-directed mutagenesis and X-ray crystallographic studies of LinB_MI_ (Okai et al. [2013]) indicated that (i) these five residues are not essential to the LinB_MI_-type activity, but they all significantly contribute to this activity, and (ii) three of the five residues, V112, L138, and I253, are more important than T81 and T135 for the conversion of PCHL. The structural basis for the importance of the seven amino acid residues of LinB_MI_ can be partially explained by analysis of its tertiary structure (Figure 2). V134 and V112 are located at the catalytic pocket near the nucleophile residue (D108) and at the bottom of the substrate binding pocket, respectively, while L138, H247, and I253 are located at the access tunnels to the catalytic pocket. Therefore, these five amino acid residues may be directly involved in LinB_MI_-type activity (Okai et al. [2013]). The effect of T135 on LinB_MI_-type activity may be due to its interaction with I253. However, it is unclear how T81, which is located at the protein surface and far from the active site, affect the activity.

**Figure 3:**
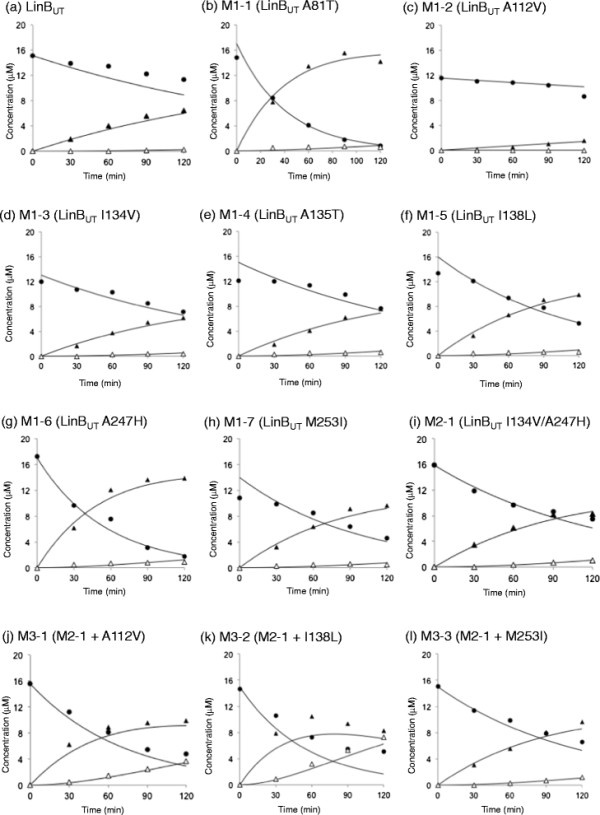
**Degradation of β-HCH in reaction mixtures containing LinB**_**UT**_**and its mutant derivatives.** LinB_UT_ wild-type **(a)** and its mutants, M1-1 **(b)**, M1-2 **(c)**, M1-3 **(d)**, M1-4 **(e)**, M1-5 **(f)**, M1-6 **(g)**, M1-7 **(h)**, M2-1 **(i)**, M3-1 **(j)**, M3-2 **(k)**, M3-3 **(l)**. The closed circle and closed and open triangles represent β-HCH, PCHL, and TCDL, respectively. Each value given is the mean of triplicates. Kinetic data were fitted to the irreversible two-step reaction scheme of β-HCH conversion to TCDL via PCHL (Scheme 1 in Materials and methods) by using GEPASI 3.2 software (Mendes [1997]) and shown in solid lines. The specificity constants and their standard errors for both reaction steps (*k*_1_ and *k*_2_) were obtained from the calculation (Table 1).

**Figure 4:**
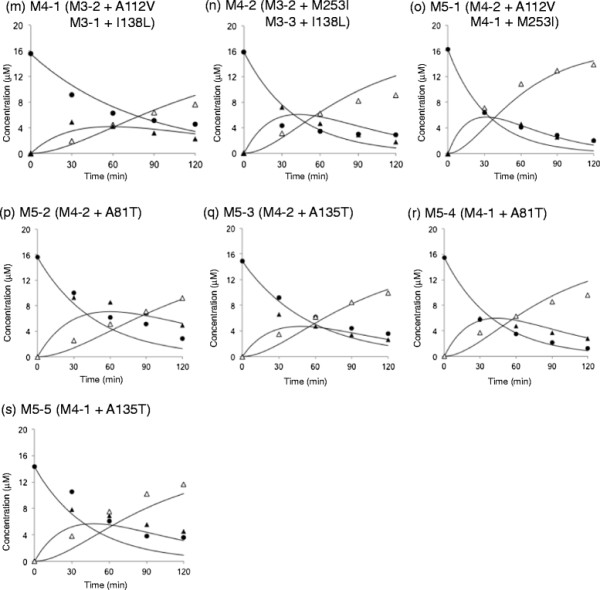
**Degradation of β-HCH in reaction mixtures containing LinB**_**UT**_**mutant derivatives.** M4-1 **(m)**, M4-2 **(n)**, M5-1 **(o)**, M5-2 **(p)**, M5-3 **(q)**, M5-4 **(r)**, and M5-5 **(s)**. See legend of Figure 3.

In this study, cumulative mutations were introduced into LinB_UT_, and the resulting intermediate mutant enzymes between LinB_UT_ and LinB_MI_ were characterized in order to gain more insight into the molecular evolution of LinB towards β-HCH degradation activity. Since the LinB_UT_ I134V/A247H (=M2-1) mutant showed only weak LinB_MI_-type activity in our previous study (Ito et al. [2007]), cumulative mutations at the positions A112, I138, and M253 were introduced into the M2-1 mutant. On the basis of kinetic analyses of the resulting three-, four-, and five-point LinB_UT_ mutants (Figures 3, 4 and Table 1), enhancement of the catalytic performance of LinB_UT_ towards β-HCH is discussed.

**Table 1:** **Specificity constants of** LinB_UT_**,LinB**_**MI**_**,and their intermediate mutants**

**Enzyme**			**Position of the different amino acid residues**							**Specificity constant** ***k***_**cat**_**/*K***_**m**_**(M**^**-1**^**s**^**-1**^**)**				**Data source**
**Fold**^**a**^	**Mutant name**		**81**	**112**	**134**	**135**	**138**	**247**	**253**	**HCH → PCHL**	**% to LinB**_**MI**_	**PCHL → TCDL**	**% to LinB**_**MI**_	
0	LinB_UT_		A	A	I	A	I	A	M	17 ± 0	9	2.0 ± 1	0.2	This study^b^
1	LinB_UT_ A81T	M1-1	T	A	I	A	I	A	M	92 ± 3	50	2.0 ± 1	0.2	This study
1	LinB_UT_ A112V	M1-2	A	V	I	A	I	A	M	4 ± 0	2.1	0	0	This study
1	LinB_UT_ I134V	M1-3	A	A	V	A	I	A	M	21 ± 0	11	5 ± 1	0.5	This study^b^
1	LinB_UT_ A135T	M1-4	A	A	I	T	I	A	M	23 ± 1	12	6 ± 2	0.6	This study
1	LinB_UT_ I138L	M1-5	A	A	I	A	L	A	M	35 ± 1	18	5 ± 1	0.5	This study
1	LinB_UT_ A247H	M1-6	A	A	I	A	I	H	M	68 ± 2	36	4 ± 1	0.4	This study^b^
1	LinB_UT_ M253I	M1-7	A	A	I	A	I	A	I	39 ± 1	21	4 ± 1	0.4	This study
2	LinB_UT_ I134V/A247H	M2-1	A	A	V	A	I	H	M	30 ± 0	16	7 ± 1	0.7	This study^b^
3	LinB_UT_ A112V/ I134V/A247H	M3-1	A	V	V	A	I	H	M	53 ± 3	28	17 ± 2	1.7	This study
3	LinB_UT_ I134V/I138L/A247H	M3-2	A	A	V	A	L	H	M	69 ± 10	36	31 ± 7	3.1	This study
3	LinB_UT_ I134V/A247H/M253I	M3-3	A	A	V	A	I	H	I	33 ± 1	17	7 ± 1	0.7	This study
4	LinB_UT_ A112V/I134V/I138L/A247H	M4-1	A	V	V	A	L	H	M	48 ± 4	25	85 ± 10	8.5	This study
4	LinB_UT_ I134V/I138L/A247H/M253I	M4-2	A	A	V	A	L	H	I	1.0 ± 0.2 x 10^2^	53	83 ± 20	8.3	This study
5	LinB_UT_ A112V/I134V/I138L/A247H/M253I	M5-1	A	V	V	A	L	H	I	1.2 ± 0.1 x 10^2^	63	1.3 ± 0.2 x 10^2^	13	This study
5	LinB_UT_ A81T/I134V/I138L/A247H/M253I	M5-2	T	A	V	A	L	H	I	79 ± 10	42	50 ± 10	5	This study
5	LinB_UT_ I134V/A135T/I138L/A247H/M253I	M5-3	A	A	V	T	L	H	I	68 ± 8	36	90 ± 20	9	This study
5	LinB_UT_ A81T/A112V/I134V/I138L/A247H	M5-4	T	V	V	A	L	H	M	91 ± 9	48	83 ± 10	8.3	This study
5	LinB_UT_ A112V/I134V/A135T/I138L/A247H	M5-5	A	V	V	T	L	H	M	84 ± 20	44	73 ± 20	7.3	This study
6	LinB_MI_ T81A	M6-1	A	V	V	T	L	H	I	68 ± 3	36	9.5 ± 5.0 x 10^2^	95	Okai et al. [2013]
6	LinB_MI_ V112A	M6-2	T	A	V	T	L	H	I	1.0 ± 0.09 x 10^2^	53	2.3 ± 0.4 x 10^2^	23	Okai et al. [2013]
6	LinB_MI_ V134I	M6-3	T	V	I	T	L	H	I	1.2 ± 0.05 x 10^2^	63	80 ± 3	8	Ito et al. [2007]
6	LinB_MI_ T135A	M6-4	T	V	V	A	L	H	I	83 ± 5	44	4.2 ± 1 x 10^2^	42	Okai et al. [2013]
6	LinB_MI_ L138I	M6-5	T	V	V	T	I	H	I	1.3 ± 0.1 x 10^2^	68	2.2 ± 0.4 x 10^2^	22	Okai et al. [2013]
6	LinB_MI_ H247A	M6-6	T	V	V	T	L	A	I	2.1 ± 0.2 x 10^2^	111	2.4 ± 0.2 x 10^2^	24	Ito et al. [2007]
6	LinB_MI_ I253M	M6-7	T	V	V	T	L	H	M	2.1 ± 0.3 x 10^2^	111	1.4 ± 0.2 x 10^2^	14	Okai et al. [2013]
7	LinB_MI_		T	V	V	T	L	H	I	1.9 ± 0.08 x 10^2^	100	1.0 ± 0.3 x 10^3^	100	Okai et al. [2013]

^a^Number of mutation into LinB_UT_.

^b^The activities of LinB_UT_, M1-3, M1-6, and M2-1 have already been measured in our previous study (Ito et al. [2007]), but they were reanalyzed in this study for the critical comparison with other mutants.

## Materials and methods

### Expression and purification of enzymes

The nomenclature of LinB mutants used in this study is shown in Table 1. An established method was used for site-directed mutagenesis (Ito et al. [2007]). The expression plasmids for the 10 LinB_UT_ multiple mutants, M3-1 (A112V/I134V/A247H), M3-2 (I134V/I138L/A247H), M3-3 (I134V/A247H/M253I), M4-1 (A112V/I134V/I138L/A247H), M4-2 (I134V/I138L/A247H/M253I), M5-1 (A112V/I134V/I138L/A247H/M253I), M5-2 (A81T/I134V/I138L/A247H/M253I), M5-3 (I134V/A135T/I138L/A247H/M253I), M5-4 (A81T/A112V/I134V/I138L/A247H), and M5-5 (A112V/I134V/A135T/I138L/A247H), were constructed as described previously using the vector pAQNM (Ito et al. [2007]). The His-tagged target proteins were expressed under the control of the *tac* promoter and *lacI*^q^. *E. coli* BL21 Star (DE3) cells expressing LinB_UT_ mutants were disrupted by bacteriolysis using a CelLytic B Reagent (Sigma), and His-tagged enzymes were purified by using BD TALON Metal Affinity Resins (BD Biosciences). Only one protein band corresponding to about 33 kDa was observed on sodium dodecyl sulfate (SDS)-polyacrylamide gel electrophoresis after the purification (data not shown).

### Enzymatic assays

The purified enzyme was incubated with 17 μM of β-HCH in 50 mM potassium phosphate buffer (pH 7.5) containing 10% (v/v) glycerol at 30°C. The enzyme concentration in the reaction mixture was 150 μg/ml. The mixture (100 μl) was extracted with an equal volume of ethyl acetate and then analyzed by a Shimadzu GC-17A gas chromatograph equipped with a ^63^Ni electron capture detector (ECD) and Rtx-1 capillary column (30 m × 0.25 mm × 0.25 μm; Restek). The column temperature was increased from 160°C to 200°C at a rate of 4°C/min, and then from 200°C to 260°C at a rate of 20°C/min. The gas flow rate was a constant 30 ml/min. As the internal standard, 10 μM 2,4,5-trichlorophenol was used. Due to the low solubility of β-HCH in water, the *k*_cat_ and *K*_m_ values of the mutants could not be calculated. Kinetic data were fitted to the irreversible two-step reaction scheme of β-HCH conversion to TCDL via PCHL (Scheme 1). Nonlinear regression provided estimates of the specificity constants and standard errors for both reaction steps (*k*_1_ and *k*_2_) by using GEPASI 3.2 software (Mendes [1997]).

(1) $$\begin{array}{l} E+{\mathrm{HCH}}^{\underset{\to }{k1}}E+{\mathrm{PCHL}}^{\underset{\to }{k2}}E+\mathrm{TCDL}\\ \;\text{Scheme}\;1 \end{array}$$

## Results

### Characterization of single- and double-point mutants of LinB_UT_

**Figure 5:**
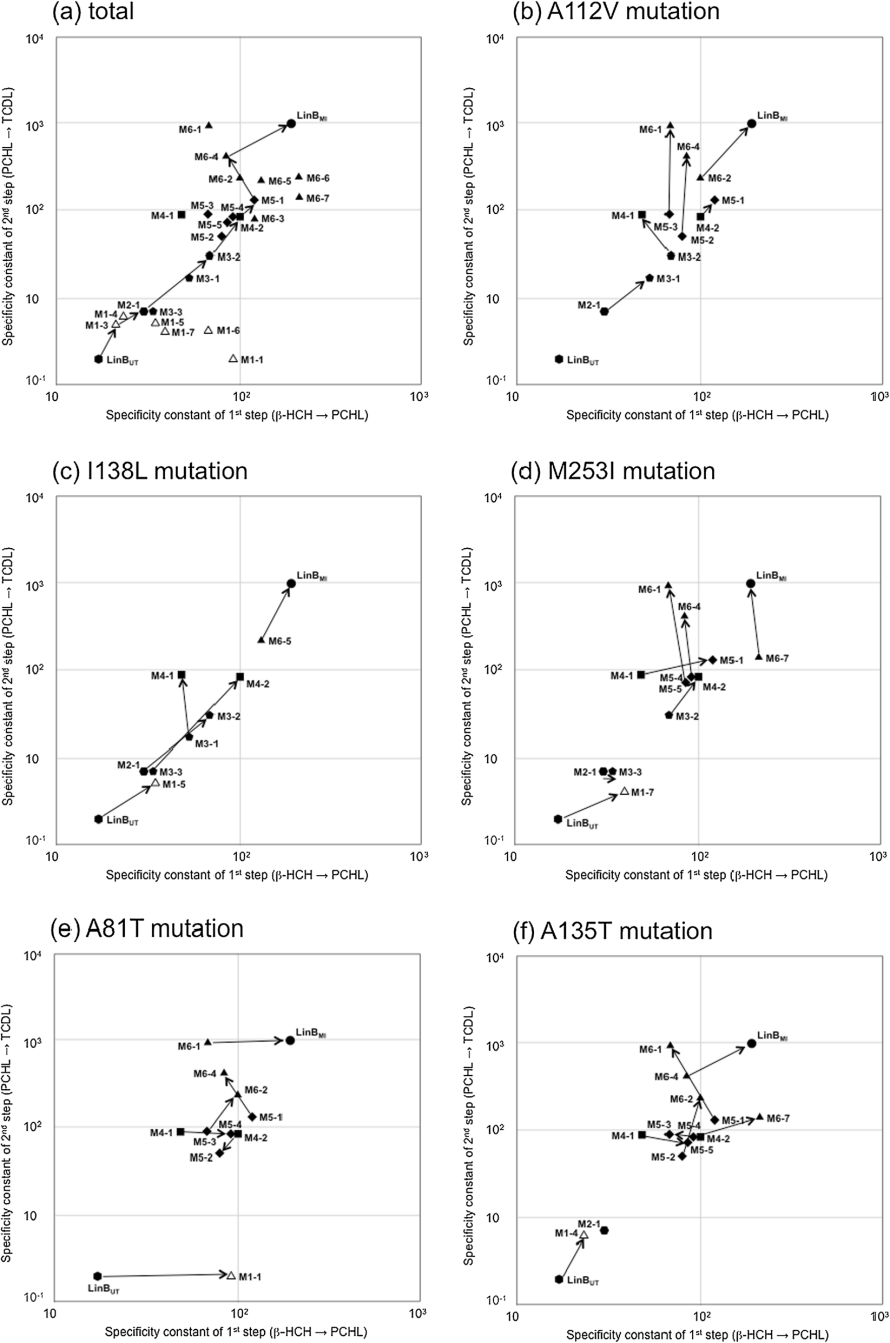
**The β-HCH degradation activities of LinB**_**UT**_**, LinB**_**MI**_**, and their intermediate mutants.** Specificity constants of LinB_UT_ (vertical hexagon), LinB_MI_ (circle), and their intermediate mutants (single, open triangle; double, horizontal hexagon; 3-point, pentagon; 4-point, square; 5-point, diamond; and 6-point, closed triangle) for the first conversion (from β-HCH to PCHL: X axis) and the second conversion (from PCHL to TCDL: Y axis) steps (Table 1) were plotted in logarithmical values. The effects of A112V **(b)**, I138L **(c)**, M253I **(d)**, A81T (**e**), and A135T **(f)** mutations were extracted from the total plot **(a)** and shown by arrows. One potential evolutionary route from LinB_UT_ to LinB_MI_ by the accumulation of seven point mutations is shown by arrows **(a)**.

Since M2-1 showed only a weak LinB_MI_-type activity in the previous study (Ito et al. [2007]), we introduced further mutations into M2-1 in this study. However, for the critical comparison with other mutants, the seven single-point mutants [A81T (M1-1), A112V (M1-2), I134V (M1-3), A135T (M1-4), I138L (M1-5), A247H (M1-6), and M253I (M1-7)] of LinB_UT_ and M2-1 were also kinetically characterized in this study, and the importance of individual mutations for LinB_MI_ activity towards β-HCH was assessed (Figure 3 and Table 1). Among the seven single-point mutations, only the A112V mutation had a negative effect on β-HCH degradation activity, while the other six mutations showed a slightly positive effect on enzymatic activity towards β-HCH (Figure 3b-h and Figure 5). Interestingly, the A81T mutation had a relatively strong effect on the first conversion (β-HCH to PCHL) step (Figure 3b and Figure 5e). The involvement of T81 in the first step was consistent with the decrease in this step by the reciprocal T81A mutation into LinB_MI_ (M6-1) (Figure 5e and Additional file 1: Figure S1b) (Okai et al. [2013]). M2-1 showed higher activity for the second conversion (PCHL to TCDL) step (Table 1) than all the single mutants, but its activity was still weak (Figure 3i and Figure 5a).

### Characterization of three-point mutants of LinB_UT_

The importance of V112, L138, and I253 for LinB_MI_-type activity was suggested in a previous study (Okai et al. [2013]). Therefore, the A112V, I138L, and M253I mutations were independently introduced into M2-1, resulting in three-point mutants: M3-1 (Figure 3j), M3-2 (Figure 3k), and M3-3 (Figure 3l), respectively. These three mutations showed different effects on β-HCH degradation activity (Figure 5a and Table 1). The I138L mutation positively influenced both conversion steps (Figure 5c: M2-1 to M3-2), while the M253I mutation had only a weak positive effect on the first conversion step (Figure 5d: M2-1 to M3-3). The A112V mutation showed positive effects on both conversion steps (Figure 5b: M2-1 to M3-1), although the effects were lower than those of I138L (Figure 5a).

### Characterization of four-point mutants of LinB_UT_

Since M3-2 showed the highest β-HCH degradation activity among the evaluated three-point mutants (Figure 5a and Table 1), the A112V and M253I mutations were independently introduced into M3-2, giving rise to four-point mutants, M4-1 (Figure 4m) and M4-2 (Figure 4n), respectively. These mutations resulted in similar levels of positive effects on the second conversion step, but the M253I (M3-2 to M4-2) and A112V (M3-2 to M4-1) mutations had positive and negative effects, respectively, on the rates of the first conversion step (Figure 5a, b, d and Table 1). M4-1 and M4-2 are equivalent to the I138L mutants of M3-1 and M3-3, respectively. Therefore, the I138L mutation had a relatively high level of positive effects on the second conversion step in the cases of M3-1 to M4-1 and M3-3 to M4-2, and also had a positive effect on the first conversion step in the latter case (Figure 5c and Table 1).

### Characterization of five-point mutants of LinB_UT_

We further constructed a five-point mutant of LinB_UT_ (A112V/I134V/I138L/A247H/M253I: M5-1) (Figure 4o), which has the mutations at all five amino acid residues that were suggested to be important for β-HCH degradation activity. This mutant can be constructed by the introduction of M253I and A112V mutations into M4-1 and M4-2, respectively. The enzymatic activities of M5-1 indeed increased in both conversion steps compared with those by the parental M4-1 and M4-2 mutants (Figure 5a and Table 1).

All of the other four possible five-point mutants from M4-2 or M4-1 were also constructed: M5-2 (Figure 4p) and M5-3 (Figure 4q) from M4-2, and M5-4 (Figure 4r) and M5-5 (Figure 4s) from M4-1. None of them surpassed the activity of M5-1 (Figure 5a). The rates of both conversion steps using M5-2 and M5-3 decreased or remained at levels similar to those of the M4-2 mutant (Figure 5a). On the other hand, the rates of the first conversion step by M5-4 and M5-5 were higher than that by the parental enzyme, M4-1, but the rates of the second conversion step of these mutants remained indistinguishable from that of M4-1 (Figure 5a).

## Discussion

In this study, cumulative substitution mutations were introduced into LinB_UT_, and various intermediate mutant enzymes between LinB_UT_ and LinB_MI_ were kinetically characterized (Figures 3, 4 and Table 1). Since LinB has promiscuous enzymatic activities towards various compounds, including other HCH isomers and various haloalkanes (Lal et al. [2010]), the functional evolution of LinB seems too complicated to analyze. However, we focused in this study on its β-HCH degradation activity as a representative model. Overall, the β-HCH degradation activities of the mutants gradually changed to those of a LinB_MI_-type enzyme according to the number of introduced mutations (Figure 5a), indicating that the function of LinB towards this activity can evolve in a stepwise manner. However, the effects of the particular amino acid substitutions depended on the order of the introduced mutations or on the combination with other mutations, and every substitution influenced both the first and second conversion steps (Figure 5b-f). Especially, the I138L mutation showed relatively strong positive effects on both conversion steps in almost all mutant enzymes examined (Figure 5c), suggesting this mutation plays a key role in LinB_MI_-type activity. At the beginning of this study, we mainly focused on three mutations, A112V, I138L, and M253I, on the basis of the results of our previous mutational and structural analyses of LinB_MI_ (Okai et al. [2013]), but our additional analysis in this study also confirmed the involvement of A81T and A135T mutations in β-HCH degradation activity (Figure 5e, f). It is of interest that the T81 residue of LinB_MI_ was mainly involved in the first conversion step (Figure 5e). However, it is at present unclear how this residue contributed so substantially to such a step, because T81 is located on the protein surface and far away from the active site and access tunnels (Figure 2) (Okai et al. [2013]).

Two examples of plausible evolutionary routes of different protein functions between two highly similar proteins have recently been reported. One example is the route to the formation of atrazine chlorohydrolase (AtzA) and melamine deaminase (TriA) (Noor et al. [2012]), which are 98% identical (nine amino acid differences in the 475 amino acid proteins). AtzA catalyzes the dehalogenation of halo-substituted triazine ring compounds but shows no activity towards melamine or ammeline (Seffernick et al. [2001]), whereas TriA has no detectable activity toward the halo-triazine substrates (Seffernick et al. [2001]). The nine amino acid substitutions for generating the different enzymatic activities could have occurred in either enzyme (Noor et al. [2012]). The other example is the route of the evolution of NtdR (a regulator of the nitrotoluene degradation pathway) from NagR (a regulator of the naphthalene degradation pathway) (Ju et al. [2009]). Although these two regulators are 98% identical (five differences among 301 amino acids), NtdR, but not NagR, can recognize a wide spectrum of nitroaromatic compounds. It has been proposed that NtdR evolved from NagR by stepwise broadening of the effector range without loss of the original function (Ju et al. [2009]). We also demonstrated in this study that LinB_UT_ could be changed to LinB_MI_ by the accumulation of seven point mutations, and that the plausible evolutionary routes could be predicted. For example, if the β-HCH degradation activity of LinB_UT_ increased under relevant selection pressure, the following order of mutations would be most likely: I134V (LinB_UT_ to M1-3) - A247H (M1-3 to M2-1) - I138L (M2-1 to M3-2) - M253I (M3-2 to M4-2) - A112V (M4-2 to M5-1) - A81T (M5-1 to M6-4) - A135T (M6-4 to LinB_MI_) (Figure 5a). In this order, the activity for the second conversion increases gradually by every mutation step. Although the activity for the first conversion decreases at the 6th step, the second conversion seems to be more important for the LinB_MI_-type activity, since LinB_UT_ has nearly no activity for the second conversion.

However, we have to keep in mind that it is impossible at present to predict the diverging processes of LinB_MI_ and LinB_UT_ in the environment, since (i) we have not constructed all possible intermediate mutants between LinB_MI_ and LinB_UT_, and (ii) their common ancestral enzyme remains unknown. All eight nucleotide substitutions (in the seven codons) between *linB*_MI_ and *linB*_UT_ are nonsynonymous (Ito et al. [2007]), suggesting that LinB_MI_ and LinB_UT_ diverged relatively recently from a common ancestral LinB protein under strong selection pressure. However, the benefit of the LinB_MI_-type activity to the host cells is still unknown. TCDL is a dead-end product in strain MI1205 (Ito et al. [2007]), indicating the inability of this strain to use β-HCH as a carbon and energy source. Furthermore, TCDL seems to be more toxic than β-HCH, because a UT26 derivative whose *linB*_UT_ gene is replaced by *linB*_MI_ showed a growth defect in the presence of β-HCH (unpublished data). In other words, the β-HCH degradation activity itself is apparently unfavorable even for the host cells in the presence of β-HCH. β-HCH degradation activity may be beneficial when cells with this activity coexist with other types of cells having enzymes for the metabolism of TCDL. In the γ-HCH metabolism, LinB converts 1,3,4,6-tetrachloro-1,4-cyclohexadiene, which is produced from γ-HCH by LinA (Nagata et al. [1993]). Our preliminary analysis indicated that there was no difference between the LinB_UT_- and LinB_MI_-catalyzed transformation activities towards the intermediate (unpublished data). It has been proposed that enzymatic promiscuity is important for protein evolution (Aharoni et al. [2005]; Khersonsky et al. [2006]), and LinB_MI_ may be in a more promiscuous state than LinB_UT_. More detailed studies are needed to elucidate the physiological significance of the activity unique to LinB_MI_.

All seven dissimilar amino acid residues are involved in the β-HCH degradation activity unique to LinB_MI_, and their positions in the structure of LinB_MI_ can partially explain their functions in this activity, as described above (Figure 2) (Okai et al. [2013]). However, the detailed mechanism by which they contribute to the catalytic activity is not fully understood, because the effect of each successive amino acid substitution depends on the combination of other mutations. Furthermore, all seven mutations showed substantial positive effects at the final stage of their introduction (six-point mutants of LinB_UT_, Figure 5a). These results suggested that the synergetic effects are important for the activity. Numerous naturally occurring LinB variants have recently been reported (Additional file 1: Table S1). Although the seven amino acid residues described herein indeed seem to be the hot spots for mutations in the variants, variations in other amino acid residues, such as 134L, 247S, and 253L, were also found (Additional file 1: Table S1). To discuss the evolution of LinB critically, the effects of such novel substitutions on the β-HCH degradation activity should be addressed in future studies. Furthermore, this study also describes the influence of several mutations on the enzymatic activity of LinB and helps in understanding the structure-function relationship. This information might be useful in future for rational design of LinBs with improved activity.

## Competing interests

The authors declared that they have no competing interests.

## Authors’ contributions

RM, HT, YNit, MIsh, and MIto designed and performed experiments. YO participated in the design of the study. JD and ZP analyzed data. MT, JD, ZP, and YNag participated in the design and coordination of this study and drafted the manuscript. YNag conceived the study and is the responsible of the entire project. All authors read and approved the final manuscript.

## Additional file

## Supplementary Material

Additional file 1: Figure S1. Degradation of β-HCH (closed circle) and appearance of its metabolites, PCHL (closed triangle) and TCDL (open triangle), in reaction mixtures containing LinB_MI_ wild-type (a), and seven point mutants of LinB_MI_ (b-h). Values given are the mean of triplicates. Kinetic data were fitted to the irreversible two-step reaction structure of β-HCH conversion to TCDL via PCHL (Scheme 1 in Materials and Methods) by using GEPASI 3.2 software (Mendes [1997]) and shown in solid lines. The specificity constants and their standard errors for both reaction steps (*k*_1_ and *k*_2_) were obtained from the calculation (Table 1). The same data were used that have already been published by Ito et al ([2007]) (panels d and g) and Okai et al ([2013]) (panels a, b, c, e, f, and h). **Table S1.** Naturally occurring LinB variants. Click here for file

## References

[B1] Aharoni A Gaidukov L Khersonsky O Mc QGS Roodveldt C Tawfik DS The ‘evolvability’ of promiscuous protein functions Nature Genet 2005 37 73 76 15568024 10.1038/ng1482

[B2] Damborsky J Koca J Analysis of the reaction mechanism and substrate specificity of haloalkane dehalogenases by sequential and structural comparisons Protein Eng 1999 12 989 998 10.1093/protein/12.11.989 10585505

[B3] Gupta A Kaushik C Kaushik A Degradation of hexachlorocyclohexane (HCH; α, β, γ and δ) by *Bacillus circulans* and *Bacillus brevis* isolated from soil contaminated with HCH Soil Biol Biochem 2000 32 1803 1805 10.1016/S0038-0717(00)00072-9

[B4] Gupta A Kaushik CP Kaushik A Degradation of hexachlorocyclohexane isomers by two strains of *Alcaligenes faecalis* isolated from a contaminated site Bull Environ Contam Toxicol 2001 66 794 800 11353383 10.1007/s001280078

[B5] Ito M Prokop Z Klvana M Otsubo Y Tsuda M Damborsky J Nagata Y Degradation of β-hexachlorocyclohexane by haloalkane dehalogenase LinB from γ-hexachlorocyclohexane-utilizing bacterium *Sphingobium* sp. MI1205 Arch Microbiol 2007 188 313 325 10.1007/s00203-007-0251-8 17516046

[B6] Janssen DB Evolving haloalkane dehalogenases Curr Opin Chem Biol 2004 8 150 159 10.1016/j.cbpa.2004.02.012 15062775

[B7] Johri A Dua M Tuteja D Saxena R Saxena D Lal R Degradation of α, β, γ and δ-hexachlorocyclohexanes by *Sphingomonas paucimobilis* Biotechnol Lett 1998 20 885 887 10.1023/A:1005323811769

[B8] Ju KS Parales JV Parales RE Reconstructing the evolutionary history of nitrotoluene detection in the transcriptional regulator NtdR Mol Microbiol 2009 74 826 843 10.1111/j.1365-2958.2009.06904.x 19849778 PMC10423642

[B9] Khersonsky O Roodveldt C Tawfik DS Enzyme promiscuity: evolutionary and mechanistic aspects Curr Opin Chem Biol 2006 10 498 508 10.1016/j.cbpa.2006.08.011 16939713

[B10] Lal R Dogra C Malhotra S Sharma P Pal R Diversity, distribution and divergence of *lin* genes in hexachlorocyclohexane-degrading sphingomonads Trends Biotechnol 2006 24 121 130 10.1016/j.tibtech.2006.01.005 16473421

[B11] Lal R Pandey G Sharma P Kumari K Malhotra S Pandey R Raina V Kohler HP Holliger C Jackson C Oakeshott JG Biochemistry of microbial degradation of hexachlorocyclohexane and prospects for bioremediation Microbiol Mol Biol Rev 2010 74 58 80 10.1128/MMBR.00029-09 20197499 PMC2832351

[B12] Mendes P Biochemistry by numbers: simulation of biochemical pathways with Gepasi 3 Trends Biochem Sci 1997 22 361 363 10.1016/S0968-0004(97)01103-1 9301339

[B13] Mohn WW Mertens B Neufeld JD Verstraete W de Lorenzo V Distribution and phylogeny of hexachlorocyclohexane-degrading bacteria in soils from Spain Environ Microbiol 2006 8 60 68 10.1111/j.1462-2920.2005.00865.x 16343322

[B14] Nagata Y Nariya T Ohtomo R Fukuda M Yano K Takagi M Cloning and sequencing of a dehalogenase gene encoding an enzyme with hydrolase activity involved in the degradation of γ-hexachlorocyclohexane in *Pseudomonas paucimobilis* J Bacteriol 1993 175 6403 6410 7691794 10.1128/jb.175.20.6403-6410.1993 PMC206747

[B15] Nagata Y Prokop Z Sato Y Jerabek P Kumar A Ohtsubo Y Tsuda M Damborsky J Degradation of β-hxachlorocyclohexane by haloalkane dehalogenase LinB from *Sphingomonas paucimobilis* UT26 Appl Environ Microbiol 2005 71 2183 2185 10.1128/AEM.71.4.2183-2185.2005 15812056 PMC1082558

[B16] Noor S Taylor MC Russell RJ Jermiin LS Jackson CJ Oakeshott JG Scott C Intramolecular epistasis and the evolution of a new enzymatic function PLoS One 2012 7 6 e39822 10.1371/journal.pone.0039822 22768133 PMC3387218

[B17] Okai M Ohtsuka J Imai LF Mase T Moriuchi R Tsuda M Nagata K Nagata Y Tanokura M Crystal structure and site-directed mutagenesis analyses of haloalkane dehalogenase LinB from *Sphingobium* sp. MI1205 J Bacteriol 2013 195 2642 2651 10.1128/JB.02020-12 23564170 PMC3676048

[B18] Phillips TM Seech AG Lee H Trevors JT Biodegradation of hexachlorocyclohexane (HCH) by microorganisms Biodegradation 2005 16 363 392 10.1007/s10532-004-2413-6 15865341

[B19] Prokop Z Monincova M Chaloupkova R Klvana M Nagata Y Janssen DB Damborsky J Catalytic mechanism of the haloalkane dehalogenase LinB from *Sphingomonas paucimobilis* UT26 J Biol Chem 2003 278 45094 45100 10.1074/jbc.M307056200 12952988

[B20] Seffernick JL de Souza ML Sadowsky MJ Wackett LP Melamine deaminase and atrazine chlorohydrolase: 98 percent identical but functionally different J Bacteriol 2001 183 2405 2410 10.1128/JB.183.8.2405-2410.2001 11274097 PMC95154

[B21] Sharma P Raina V Kumari R Malhotra S Dogra C Kumari H Kohler HP Buser HR Holliger C Lal R Haloalkane dehalogenase LinB is responsible for β- and δ-hexachlorocyclohexane transformation in *Sphingobium indicum* B90A Appl Environ Microbiol 2006 72 5720 5727 10.1128/AEM.00192-06 16957186 PMC1563659

[B22] Vijgen J Abhilash PC Li YF Lal R Forter M Torres J Singh N Yunus M Tian C Schaffer A Weber R Hexachlorocyclohexane (HCH) as new Stockholm Convention POPs-a global perspective on the management of Lindane and its waste isomers Environ Sci Pollut Res Int 2011 18 152 162 10.1007/s11356-010-0417-9 21104204

[B23] Willett KL Ulrich EM Hites RA Differential toxicity and environmental fates of hexachlorocyclohexane isomers Environ Sci Technol 1998 32 2197 2207 10.1021/es9708530

