# Supplementary material for: Stepwise enhancement of catalytic performance of haloalkane dehalogenase LinB towards β-hexachlorocyclohexane
Source: AMB Express. 2014 Sep 21;4:72. doi: 10.1186/s13568-014-0072-5 (PMC4230811; doi:10.1186/s13568-014-0072-5)
Supplement: Additional file 1: Figure S1. — Degradation of β-HCH (closed circle) and appearance of its metabolites, PCHL (closed triangle) and TCDL (open triangle), in reaction mixtures containing LinBMI wild-type (a), and seven point mutants of LinBMI (b-h). Values given are the mean of triplicates. Kinetic data were fitted to the irreversible two-step reaction structure of β-HCH conversion to TCDL via PCHL (Scheme 1 in Materials and Methods) by using GEPASI 3.2 software (Mendes [1997]) and shown in solid lines. The specificity constants and their standard errors for both reaction steps (k1 and k2) were obtained from the calculation (Table 1). The same data were used that have already been published by Ito et al ([2007]) (panels d and g) and Okai et al ([2013]) (panels a, b, c, e, f, and h). Table S1. Naturally occurring LinB variants. [file s13568-014-0072-5-S1.doc]

**Supplemental Materials for**

**Stepwise enhancement of catalytic performance of haloalkane dehalogenase LinB towards β-hexachlorocyclohexane**

Ryota Moriuchi,^1$^ Hiroki Tanaka,^1^ Yuki Nikawadori,^1^ Mayuko Ishitsuka,^1^ Michihiro Ito, ^1#^ Yoshiyuki Ohtsubo, ^1^ Masataka Tsuda, ^1^ Jiri Damborsky, ^2^ Zbynek Prokop,^2^ and Yuji Nagata^1^*

^1^ Department of Environmental Life Sciences, Graduate School of Life Sciences, Tohoku University, Sendai 980-8577, Japan, and ^2^ Loschmidt Laboratories, Department of Experimental Biology and Research Centre for Toxic Compounds in the Environment, Faculty of Science, Masaryk University, Kamenice 5/A13, 625 00 Brno, Czech Republic

^$^ Present address: The United Graduate School of Agricultural Science, Gifu University 1-1 Yanagido, Gifu 501-1193, Japan.

^#^ Present address: Consolidated Research Institute for Advanced Science and Medical Care, Waseda University, 2-2 Wakamatsu-cho, Shinjuku, Tokyo, 162-8480, Japan

* Address correspondence to Yuji Nagata, Department of Environmental Life Sciences, Graduate School of Life Sciences, Tohoku University, 2-1-1 Katahira, Sendai, 980-8577, Japan. Tel./fax: +81-22-217-5682; e-mail: aynaga@ige.tohoku.ac.jp

**Fig. S1** Degradation of β-HCH (closed circle) and appearance of its metabolites, PCHL (closed triangle) and TCDL (open triangle), in reaction mixtures containing LinB_MI_ wild-type (a), and seven point mutants of LinB_MI_ (b-h). Values given are the mean of triplicates. Kinetic data were fitted to the irreversible two-step reaction structure of β-HCH conversion to TCDL via PCHL (Scheme 1 in Materials and Methods) by using GEPASI 3.2 software (Mendes 1997) and shown in solid lines. The specificity constants and their standard errors for both reaction steps (*k*_1_ and *k*_2_) were obtained from the calculation (Table 1). The same data were used that have already been published by Ito et al (2007) (panels d and g) and Okai et al (2013) (panels a, b, c, e, f, and h).


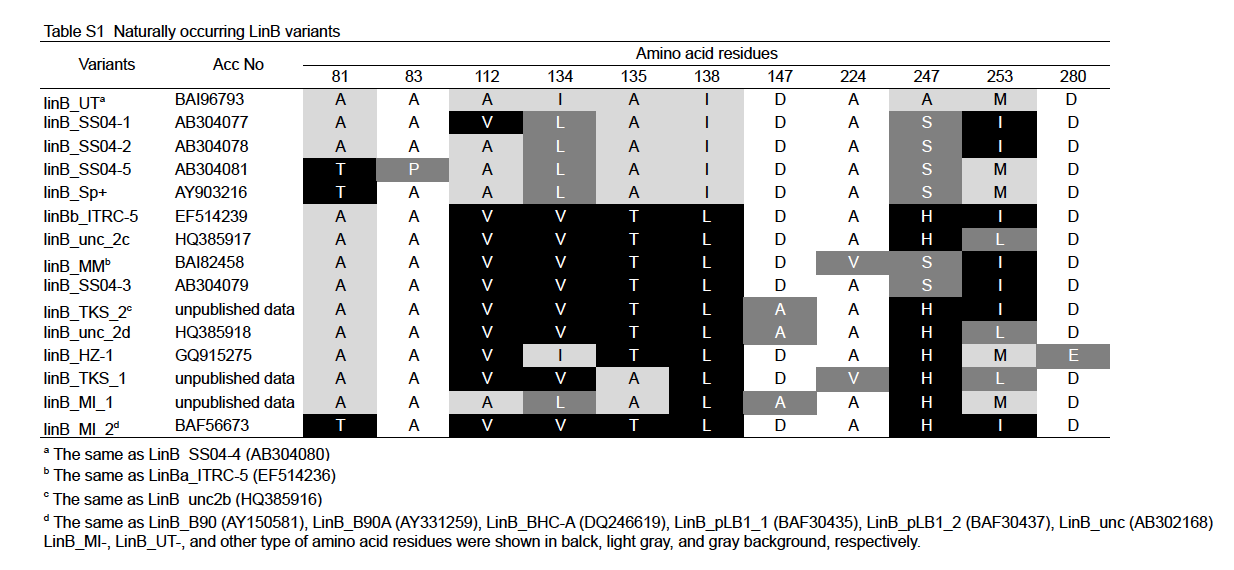


**References**

Ito M, Prokop Z, Klvana M, Otsubo Y, Tsuda M, Damborsky J, Nagata Y (2007) Degradation of β-hexachlorocyclohexane by haloalkane dehalogenase LinB from γ-hexachlorocyclohexane-utilizing bacterium *Sphingobium* sp. MI1205. Arch Microbiol 188:313-25

Mendes P (1997) Biochemistry by numbers: simulation of biochemical pathways with Gepasi 3. Trends Biochem Sci 22:361–363

Okai M, Ohtsuka J, Imai LF, Mase T, Moriuchi R, Tsuda M, Nagata K, Nagata Y, Tanokura M (2013) Crystal structure and site-directed mutagenesis analyses of haloalkane dehalogenase LinB from *Sphingobium* sp. MI1205. J Bacteriol　195:2642-2651
